# COVID‐19 Vaccination Is Not Associated With the Development of Idiopathic Inflammatory Myositis in US Veterans

**DOI:** 10.1002/acr.70023

**Published:** 2026-03-13

**Authors:** Caleb Hernández, Naomi Schlesinger, Jorge Rojas, Jessica A. Walsh, Tawnie J. Braaten, Gary A. Kunkel, Makoto Jones, Brian C Sauer, Julio Facelli, Grant W. Cannon, Dorota Lebiedz‐Odrobina

**Affiliations:** ^1^ University of Utah Salt Lake City; ^2^ George E. Wahlen VA Medical Center Salt Lake City Utah; ^3^ Puget Sound VA Medical Center Seattle Washington

## Abstract

**Objective:**

Several case reports have proposed a potential association between COVID‐19 vaccination and the subsequent development of idiopathic inflammatory myositis (IIM). This study examined prior COVID‐19 vaccination in US veterans who developed new‐onset IIM compared to those without new‐onset IIM.

**Methods:**

For this case‐control study, new‐onset incident cases of IIM were veterans enrolled in the Veterans Health Administration (VHA) with at least two IIM *International Classification of Diseases* (ICD) codes, at least one year of VHA enrollment before the first IIM ICD code, and chart review confirming incident IIM. Each IIM incident case was matched 1:5 to control patients without IIM who had similar age, sex, race, specialty clinic visits for the first IIM diagnostic code, and year of specialty clinic visit.

**Results:**

The 89 patients with new‐onset incident IIM identified were matched to 445 controls without IIM. Seven (7.9%) case patients and 29 (6.5%) control patients received their first COVID‐19 vaccination within 30 days before the index date (odds ratio [OR] 1.22, *P* = 0.643; adjusted OR 1.12, *P* = 0.657), and 11 (12.4%) case patients and 68 (15.3%) control patients received their first vaccination within 90 days of the index date (OR 0.78, *P* = 0.479; adjusted OR 0.74, *P* = 0.402). Multiple other comparisons also failed to identify a statistically significant association between COVID‐19 vaccination and IIM.

**Conclusion:**

This study is the first to compare the risk of developing myositis after receiving COVID‐19 vaccination to that of a control population. This comparison did not identify a risk for developing IIM after COVID‐19 vaccination.

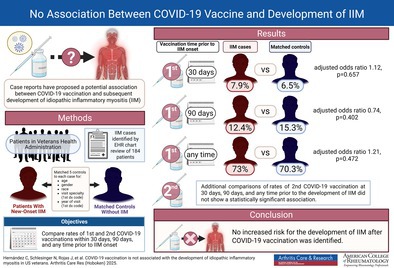

## INTRODUCTION

Multiple case reports and case series of idiopathic inflammatory myositis (IIM) following COVID‐19 vaccination have been published. Many of these cases were anti–melanoma differentiation‐associated gene 5 (anti‐MDA5) dermatomyositis,^1^, ^2^, ^3^, ^4^, ^5^, ^6^ although other types of IIM have also been reported.^7^, ^8^, ^9^ Potential mechanisms through which COVID‐19 vaccination could induce IIM have been proposed.^6^ One potential mechanism is a type I interferonopathy. The two vaccine formulations, messenger RNA (mRNA) encoding the SARS‐CoV‐2 spike (S) protein encapsulated in lipid nanoparticles or adenovirus (AdV) vectors encoding the S protein, induce production of high levels of S protein. Additionally, the innate sensors are triggered by the intrinsic adjuvant activity of the vaccines, resulting in production of type I interferon, proinflammatory cytokines, and chemokines. The RNA sensors activated by the mRNA vaccines include Toll‐like receptor 7 (TLR7) and MDA5. TLR9 is the major double‐stranded DNA sensor for the AdV vaccine.^10^ Given these theoretical concerns, there is an important need to investigate the potential for COVID‐19 vaccination to induce IIM.

**SIGNIFICANCE & INNOVATIONS**

- Case reports have hypothesized that there may be an association between the COVID‐19 vaccine and the development subsequent idiopathic inflammatory myositis (IIM).
- There are no controlled studies published that address this issue.
- We conducted a case‐control trial that did not demonstrate an association between prior COVID‐19 vaccination and onset of IIM within 30 days.
- The data may assist providers in talking to patients about the risks and benefits of COVID‐19 immunization in the context of IIM.

Evidence to date has been limited to these case reports suggesting that COVID‐19 vaccine administration may present a risk for IIM, particularly in immunocompromised patients with autoimmune diseases. There is a critical need for investigations with appropriate control populations to evaluate for the possible association between COVID‐19 vaccination and the subsequent development of IIM.

To address these concerns, an analysis of US veterans enrolled in the Veterans Health Administration (VHA) was conducted to investigate the association of COVID‐19 immunization with the subsequent development of IIM. Our objective was to conduct a case‐control study to compare the COVID‐19 vaccination rates in patients with new‐onset IIM to the COVID‐19 vaccination rates in a matched control population without IIM. This study is the first analysis to evaluate the risk of developing IIM after receiving the COVID‐19 vaccination compared to a control population.

## PATIENTS AND METHODS

### Study design and data sources

This case‐control study evaluated all US veterans enrolled in the nationwide VHA. The VHA Corporate Data Warehouse provided demographic and immunization data.^11^ Demographic data included patient age, sex, race, ethnicity, *International Classification of Diseases* (ICD) codes, duration of VHA enrollment, site of care, and smoking history. The Compensation and Pension Record Interchange was used to review electronic health record (EHR) clinic notes and laboratory data.^12^ Our research was approved by the University of Utah Office of Research Integrity and Compliance (IRB 00012917, title: Veterans Affairs Rheumatoid Arthritis Registry).

### Case patient identification

**Figure 1:**
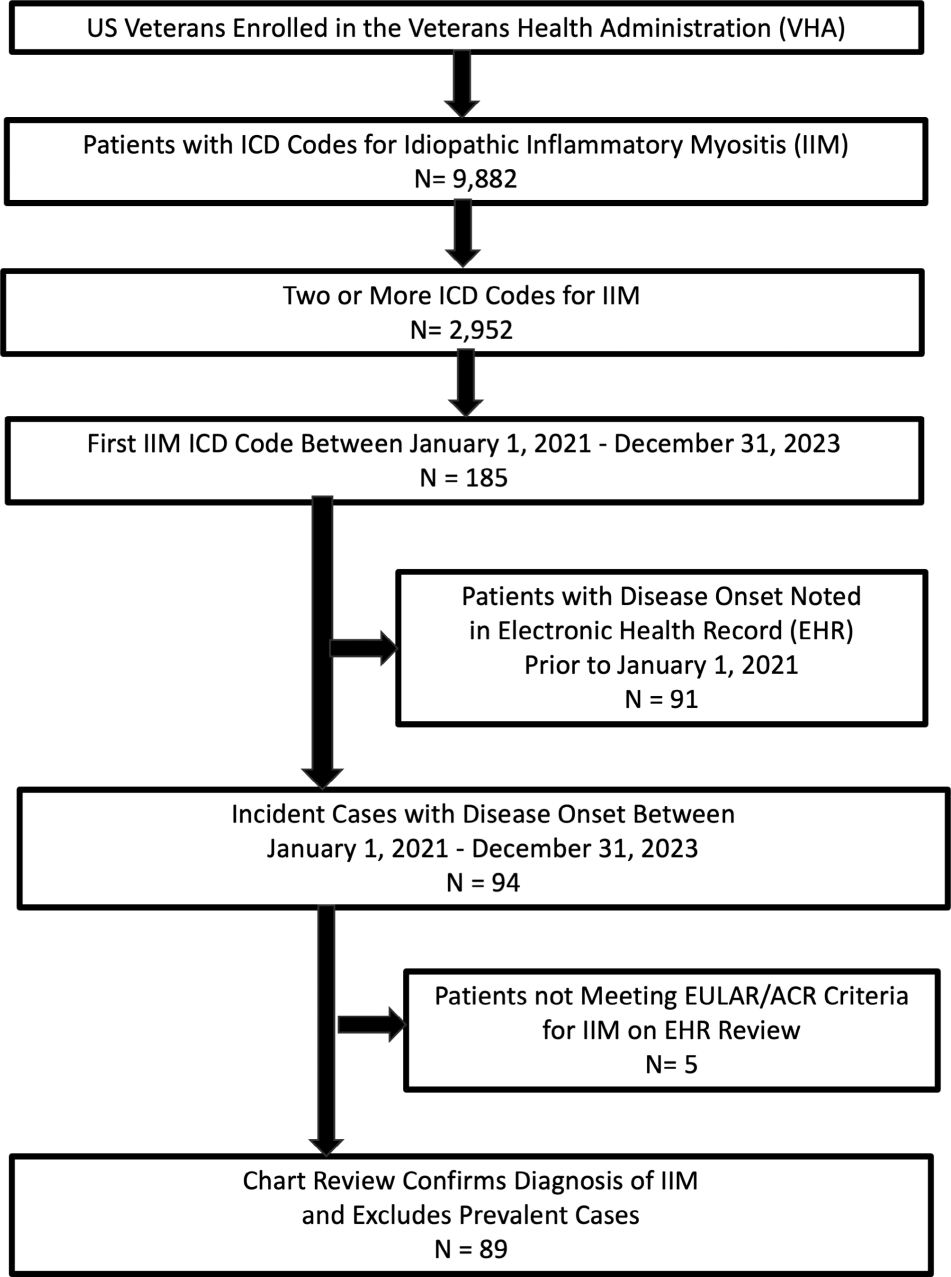
Study design schema. ACR, American College of Rheumatology; ICD, *International Classification of Diseases*.

New‐onset cases of IIM were identified through an analysis of administrative data followed by an EHR chart review (Figure 1). All veterans with either ICD‐9 or ICD‐10 codes for inflammatory myositis (Table 1) on two separate visits in the VHA, separated by at least 30 days, and who had at least one year of enrollment in the VHA before the first IIM ICD code were identified. Of this subset, patients with initial clinic visits with an IIM ICD code between January 1, 2021, and December 31, 2023, were evaluated by EHR chart review. The EHR was reviewed to (1) determine if the patient was diagnosed with IIM according to 2017 EULAR/American College of Rheumatology (ACR) criteria for IIM,^13^ and, if IIM was diagnosed, then the IIM subgroup by EULAR/ACR criteria was recorded; (2) determine if the patient had new‐onset incident IIM during VHA care; and (3) identify the date of IIM disease onset. The EHR review ensured that EULAR/ACR criteria for IIM^13^ were documented for all patients diagnosed within the VHA in order for them to be included in our analysis. For cases diagnosed outside the VA, if the VA provider clearly documented an IIM diagnosis by a specialist outside the VA, if the provider assessment documented IIM, and if the treatment plan was consistent with an IIM diagnosis, then the patient was included as an IIM case. Cases of inclusion body myositis were excluded from our study.

**Table 1:** ICD‐9 and ICD‐10 codes used to identify patients diagnosed with idiopathic inflammatory myositis*

Code	Description
359.6	Symptomatic inflammatory myopathy in diseases classified elsewhere
710.3	Dermatomyositis
710.4	Polymyositis
M33.10	Other dermatomyositis, organ involvement unspecified
M33.11	Other dermatomyositis with respiratory involvement
M33.12	Other dermatopolymyositis with myopathy
M33.13	Other dermatomyositis without myopathy
M33.19	Other dermatopolymyositis with other organ involvement
M33.20	Polymyositis, organ involvement unspecified
M33.21	Polymyositis with respiratory involvement
M33.22	Polymyositis with myopathy
M33.29	Polymyositis with other organ involvement
M36.0	Dermato(poly)myositis in neoplastic disease

* ICD, *International Classification of Diseases*.

The index date was the date of IIM disease onset, identified by EHR review. Patients with new‐onset IIM in the VHA between January 1, 2021, and December 31, 2023, were included as cases in this analysis.

### Control patient assignment

Each IIM case was matched to five controls. All control patients were required to have no ICD codes for IIM at any time during VHA care. Controls were required to have at least one year of enrollment in the VHA before the IIM onset date of their matched case. Control patients were exactly matched to the corresponding case patient on sex, race, and the specialty/primary care clinic type in which the IIM diagnosis was first established. Control patients were matched to case patients by year of birth within two years. All controls were also matched to have a first specialty clinic visit in the same specialty clinic in which their index case was first diagnosed. The specialty clinic visit for these matched controls was within three months of the index case specialty clinic visit at which the case IIM diagnosis was documented. The index date for each control was the index date for IIM onset of the case to which the controls were matched.

### Exposure

The exposure in our analysis was COVID‐19 vaccination. For each patient, all COVID‐19 vaccinations within the VHA and all COVID‐19 vaccinations reported by the patient from outside the VHA were identified. For each vaccination episode, the date of vaccination and type of vaccination were recorded. Our primary objective was the analysis of the first COVID‐19 vaccination within 30 days before IIM onset. Secondary objectives analyzed expanded exposure windows before the first vaccination of both 90 days and any time before IIM onset. These three time windows, within 30 days, 90 days, and any time before IIM onset, were subsequently analyzed for the second vaccination and any COVID‐19 vaccination.

### Statistical analysis

Continuous variables are reported as mean and SD, with comparisons between groups by Student's *t*‐test. Categorical variables are reported as numbers and percentages and compared using Fisher's exact test. Associations between vaccination status and development of IIM were determined by conditional logistical regression analysis and are reported as odds ratios (ORs) with 95% confidence intervals. ORs are reported for the crude analysis, adjusted ORs are reported for the following covariates: duration of VHA enrollment, ethnicity, smoking history, and geographic region of VHA care. Analyses were performed using STATA statistical software version 18.0.

## RESULTS

### Case identification

We initially identified 9,882 veterans with the IIM codes noted in Table 1. Of that group, 2,952 met our criteria as potential incident cases with two or more IIM codes and enrollment in the VHA for at least one year before the first IIM code. Of those patients, 185 had their first IIM code in either 2021, 2022, or 2023. There were 96 patients excluded during individual chart EHR review. There were 91 patients excluded who had the onset of IIM symptoms before January 1, 2021. Of the remaining 94 patients with onset of symptoms after January 1, 2021, 5 patients who did not meet EULAR/ACR criteria for IIM^13^ were excluded. The remaining 89 patients were confirmed cases of incident IIM for the study (Figure 1).

### Patient characteristics and vaccination data

Case and control patients were well matched for age, sex, race, visit specialty with the first diagnostic code, and year of visit with the first diagnostic code (Table 2). The mean age was 62 years old, 78.7% of patients were men, 60.7% were White, 25% were Black, 39.3% of cases were diagnosed in a rheumatology clinic, and 24.5% were diagnosed in a dermatology clinic. Most IIM cases had IIM onset in 2021, with 50 (56.2%) cases diagnosed during this year. Patients were also very similar for parameters that were not included in matching criteria, including smoking status and geographic region, which showed no statistically significant differences between cases and controls. Although duration of VA enrollment before the index date was slightly longer in controls in comparison to cases (13.5 vs 12.0 years; *P* = 0.016), this statistically significant difference was not considered to be of clinical importance.

**Table 2:** Demographic and clinical information in case and control patients*

	Case (n = 89)	Control (n = 445)
Data on which patients were matched		
Age, mean ± SD, y	62.5 ± 12.3	62.4 ± 12.2
Sex, n (%)		
Male	70 (78.7)	350 (78.7)
Female	19 (21.3)	95 (21.3)
Race, n (%)		
Asian	2 (2.2)	10 (2.2)
Black	23 (25.8)	115 (25.8)
Native Hawaiian	1 (1.1)	5 (1.1)
Unknown	9 (10.1)	45 (10.1)
White	54 (60.7)	270 (60.7)
Visit with first idiopathic inflammatory myositis diagnostic code, n (%)		
Dermatology	22 (24.7)	110 (24.7)
Pulmonary	4 (4.5)	20 (4.5)
Rheumatology	35 (39.3)	175 (39.3)
Neurology	6 (6.7)	30 (6.7)
Primary care	22 (24.7)	110 (24.7)
Year of visit when first idiopathic inflammatory myositis diagnostic code was recorded in electronic health record, n (%)		
2021	33 (37.1)	166 (37.3)
2022	47 (52.8)	235 (52.8)
2023	9 (10.1)	44 (9.9)
Parameters not included in matching		
Smoking status, n (%)		
Current smoker	17 (19.1)	65 (14.6)
Former smoker	19 (21.3)	87 (19.6)
Never smoker	30 (33.7)	115 (25.8)
Unknown	23 (25.8)	178 (40.0)
Geographic region, n (%)		
Midwest	19 (21.3)	78 (17.5)
Northeast	9 (10.1)	76 (17.1)
South	40 (44.9)	186 (41.8)
West	21 (23.6)	105 (23.6)
Duration of VHA enrollment		
Years enrolled, mean ± SD	12 ± 6.4	13.5 ± 6.1
Vaccination data, n (%)		
First vaccination type (any time before June 1, 2024)		
AstraZeneca	2 (0.4)	–
Janssen	3 (3.4)	21 (4.7)
Moderna	31 (34.8)	154 (34.6)
Pfizer	40 (44.9)	191 (42.9)
No history	15 (16.9)	77 (17.3)

* VHA, Veterans Health Administration.

Overall, the COVID‐19 vaccination experience was similar in cases and controls. One or more COVID‐19 vaccination episodes were documented in 83.1% of cases and 82.7% of controls. This is similar to national vaccination rates of 81% of US citizens, with at least one dose of the COVID‐19 vaccine by May 2023.^14^ The type of COVID‐19 vaccination received was also similar between the two groups (Table 2).

### Myositis diagnosis and antibody prevalence

In IIM cases, 46 (51.6%) of patients were diagnosed with dermatomyositis, 16 (18%) were diagnosed with polymyositis, 15 (16.9%) were diagnosed with amyopathic dermatomyositis, 9 (10.1%) were diagnosed with antisynthetase syndrome, and 3 (3.4%) were diagnosed with necrotizing myositis (Table 3). The majority of IIM cases (n = 50, 56.2%) were diagnosed in 2021. The most prevalent myositis‐specific antibodies identified were transcriptional intermediary factor 1γ (TIF‐1γ) (12.4%), followed by Mi‐2 antibodies targeting the Mi‐2 nuclear antigen (10.1%) and Jo‐1 (9.0%). MDA5 antibody was identified in 3.4% of patients.

**Table 3:** Specific idiopathic inflammatory myositis diagnosis, year of diagnosis, and myositis autoantibodies in cases (n = 89)

	n (%)
Specific myositis diagnosis	
Dermatomyositis	46 (51.6)
Polymyositis	16 (18.0)
Amyopathic dermatomyositis	15 (16.9)
Antisynthetase syndrome	9 (10.1)
Necrotizing myositis	3 (3.4)
Year of idiopathic inflammatory myositis onset	
2021	50 (56.2)
2022	37 (41.6)
2023	2 (2.2)
Myositis‐specific and associated antibodies in individual patients	
TIF‐1	11 (12.4)
Mi‐2	9 (10.1)
Jo‐1	8 (9.0)
PL‐12	4 (4.5)
MDA5	3 (3.4)
NXP‐2	3 (3.4)
SAE	3 (3.4)
HMG	2 (2.2)
PM/Scl‐100	2 (2.2)
SSA	2 (2.2)
U1 RNP	2 (2.2)
ANA	1 (1.1)
Both HMG and NXP‐2	1 (1.1)
Ku	1 (1.1)
OJ	1 (1.1)
PL‐7	1 (1.1)
SAF‐1	1 (1.1)
Negative results for antibodies^a^	19 (21.3)
Not available^b^	15 (16.9)

^a^ Laboratory data available and results negative.

^b^ No laboratory results available for review.

### Association between IIM diagnosis and COVID‐19 vaccination

Our primary analysis showed that there were 7 (7.9%) IIM case patients and 29 (6.5%) control patients who received their first vaccination within 30 days before the index date (OR 1.22, *P* = 0.643; adjusted OR 1.12, *P* = 0.657) (Table 4). In expanding our analysis to include additional COVID‐19 exposure intervals before IIM onset, there were 11 (12.4%) case patients and 68 (15.3%) control patients who had received their first vaccination within 90 days of the index date (OR 0.78, *P* = 0.479; adjusted OR 0.74, *P* = 0.402) and 65 (73%) case patients and 313 (70.3%) control patients who had received their first vaccination any time before the index date (OR 1.14, *P* = 0.610; adjusted OR 1.21, *P* = 0.472). Additional comparisons with the second vaccination and any vaccination at 30 days, 90 days, and any time before the index date also failed to identify a statistically significant association of COVID‐19 vaccination with the development of IIM (Table 4).

**Table 4:** Association between development of idiopathic inflammatory myositis and COVID‐19 immunization*

	Case, n (%)	Control, n (%)	Crude odds ratio (95% CI)	*P* value	Adjusted^a^ odds ratio (95% CI)	*P* value
First COVID‐19 immunization						
Within 30 days of index date						
Yes	7 (7.9)	29 (6.5)	1.22 (0.44–2.99)	0.643	1.24 (0.48–3.18)	0.655
No	82 (92.1)	416 (93.5)	–	–	–	–
Within 90 days of index date						
Yes	11 (12.4)	68 (15.3)	0.78 (0.36–1.58)	0.479	0.70 (0.31–1.55)	0.378
No	78 (87.6)	377 (84.7)	–	–	–	–
Any time before index date						
Yes	65 (73.0)	313 (70.3)	1.14 (0.67–1.99)	0.610	1.04 (0.56–1.92)	0.900
No	24 (27.0)	132 (29.7)	–	–	–	–
Second COVID‐19 immunization						
Within 30 days of index date						
Yes	4 (4.5)	21 (4.7)	0.95 (0.23–2.92)	0.927	1.01 (0.31–3.29)	0.985
No	85 (95.5)	424 (95.3)	–	–	–	–
Within 90 days of index date						
Yes	8 (9.0)	44 (9.9)	0.90 (0.35–2.03)	0.794	0.92 (0.37–2.33)	0.873
No	81 (91.0)	401 (90.1)	–	–	–	–
Any time before index date						
Yes	60 (67.4)	309 (69.4)	0.91 (0.55–1.54)	0.706	0.74 (0.42–1.27)	0.275
No	29 (32.65)	136 (30.6)	–	–	–	–
Any COVID‐19 immunization						
Within 30 days of onset index date						
Yes	9 (10.1)	48 (10.8)	0.93 (0.39–2.02)	0.851	1.00 (0.42–2.31)	0.981
No	80 (89.9)	397 (89.2)	–	–	–	–
Within 90 days of onset index date						
Yes	15 (16.9)	81 (18.2)	0.91 (0.46–1.70)	0.762	0.89 (0.45–1.77)	0.745
No	74 (83.1)	364 (81.8)	–	–	–	–

* CI, confidence interval.

^a^ Adjusted for Veterans Health Administration enrollment duration, ethnicity, smoking history, and geographic region.

## DISCUSSION

Our case‐control study did not identify an increased risk of developing IIM after COVID‐19 vaccination among veterans in this nationwide analysis. This study is the first to compare the risk of developing IIM after receiving the COVID‐19 vaccination compared to a control population. The observation that there was no association between COVID‐19 vaccination and the development of IIM was consistent across different COVID‐19 exposure windows and the number of vaccination episodes.

Prior observational studies report the development of IIM within 3 to 30 days of vaccination.^6^, ^7^, ^15^ These studies included cases following both first and second vaccine administration. Furthermore, some studies included cases after COVID‐19 infection in addition to COVID‐19 vaccination. One study by Diaz‐Menindez et al evaluated 98 patients with new‐onset incident IIM or flares of established IIM and reported 12 (12.2%) cases with either COVID‐19 infection or vaccination.^15^ Our study was limited to only new IIM cases. This study reported new cases of dermatomyositis at 54%, which was similar to our new IIM cases of dermatomyositis at 51.6%.^15^ The reported prior COVID‐19 exposure rate was similar to our reported 6.7% and 14.7% COVID‐19 immunization exposure in combined cases and controls at 30 and 90 days, respectively, suggesting that COVID‐19 immunization and COVID‐19 infection are common events that can occur before IIM onset, without necessarily having a causal effect.

Myositis‐specific antibodies are of insufficient prevalence to allow for the analysis of specific subsets in evaluating COVID‐19 immunization and IIM. The most common antibody we identified in our cases was TIF‐1γ (12.4%), followed by Mi‐2 (10.1%) and Jo‐1 (9.0%). MDA5 appeared to be more frequently reported in other studies compared to other myositis‐specific antibodies than what we observed in our study. The study by David et al observed an increase in MDA5 positivity in Yorkshire, United Kingdom, between 2018 and 2022. Those patients, however, were predominantly female and had been exposed to both COVID‐19 infection and vaccination.^16^ In other studies, the identified cases were amyopathic dermatomyositis (ADM) or were associated with development of interstitial lung disease (ILD).^2^, ^3^, ^6^, ^7^ This should not come as a surprise, as the relation between MDA5 antibodies and ADM and ILD has been previously established.^17^ Conversely, the most common type of IIM we identified was classic dermatomyositis (51.6%). One study found a higher proportion of patients with anti‐TIF‐1γ antibodies to be over the age of 60 years.^17^ The prevalence of TIF‐1γ among our cases could be explained by the higher average age in our veteran population.

Similar to other prior case reports, the vast majority of our cases and controls were vaccinated with mRNA vaccines, namely Pfizer or Moderna.^3^, ^6^, ^7^, ^15^, ^18^ This mRNA vaccine predominance is consistent with vaccinations in all veterans, which predominantly have been with mRNA vaccines.^19^ We did, however, capture 5 cases and 21 controls vaccinated with the non‐mRNA vaccines, including the Janssen vaccine (3 cases, 21 controls) and the AstraZeneca vaccine (2 cases).

The 89 incidence cases we identified in this three‐year study period is consistent with prior work that has reported the incidence of IIM to be between 0.2 and 2 cases per 100,000 patients per year.^20^ Although this study was not designed to rigorously estimate the incidence of IIM in the VHA, the 89 cases over three years in a population of 9,100,000 veterans enrolled in the VHA would give a crude incident rate of 0.33 case per year, similar to the reported rate.^21^ Although this incidence estimate suggests that we had captured incident IIM cases in the expected range, the incident rate estimate did not include several factors, including age adjustment and specific VHA enrollment criteria.

Strengths of our study were the use of the VHA, a large nationwide cohort with access to a uniform EHR to allow the confirmation of IIM diagnosis and onset date for each case through chart review. Comprehensive vaccination data were available in the VHA, including both COVID‐19 immunization administered within the VHA and the report of COVID‐19 immunization outside the VHA. We evaluated several exposure scenarios, including cases after both the first and second vaccines at 30 days, 90 days, and any time after vaccination.

There were also some significant limitations to the study. Among our cohort, patients were predominately White men and in their sixth decade of life. The VHA serves primarily elderly male patients, which may limit the generalization of these findings to a broader population. In our study, 25.8% of patients were Black and 21.3% were female. Other reviews have identified a higher incidence of IIM in female patients, which may be difficult to compare in our study, as most veterans are male.^22^, ^23^ Also, the VHA only serves approximately 50% of US veterans, thus limiting the ability to apply these findings to all veterans. We also recognized that there are limits in applying these findings to the general population. Our study was limited to the evaluation of COVID‐19 vaccine exposure; we did not include COVID‐19 infection. Although we feel confident that IIM is not associated with COVID‐19 vaccination, we could not draw conclusions on the potential association of COVID‐19 infection with the subsequent development of IIM. Despite our efforts to match the key risk factors for IIM and adjust for other potential confounders, residual confounding is still possible. We recognize that some IIM cases in the VHA may not have been detected if specific IIM ICD codes were not used. Although the use of other nonspecific myositis codes may have detected some additional cases, we feel confident that our case identification process and validation of the diagnosis produced a sufficient number of valid IIM cases for our case‐control study. Although our results looking at the development of IIM at any time point after immunization did not note an association with durations greater than 90 days, the potential that IIM could develop several months or years after COVID‐19 immunization cannot be excluded by this work because our observation window for the development of IIM was limited to December 31, 2023. Our results could also be limited by recall bias, as patients who were diagnosed with IIM could be more likely to report vaccination outside the VA versus the control patients. Some COVID‐19 immunization episodes outside the VA may not have been captured. The collection of laboratory data and myositis‐specific antibodies was not consistent across all patients and particularly when the diagnosis was made by a specialty consultant outside the VA. In many cases, laboratory data are recorded in text notes and scanned documents, which did not allow retrieval as digital data in the EHR.

This study is the first to report the risk of developing myositis after receiving the COVID‐19 vaccination compared to a control population. No increased risk for the development of IIM after COVID‐19 vaccination was identified. Additional investigations studying the relationship between COVID‐19 vaccination and the subsequent development of IIM in different populations would be helpful in validating these results. These data provide useful information for discussion with patients when considering the risks and benefits of COVID‐19 vaccination to address vaccine misinformation and to reduce hesitancy around vaccinations.

## AUTHOR CONTRIBUTIONS

All authors contributed to at least one of the following manuscript preparation roles: conceptualization AND/OR methodology, software, investigation, formal analysis, data curation, visualization, and validation AND drafting or reviewing/editing the final draft. As corresponding author, Dr Lebiedz‐Odrobina confirms that all authors have provided the final approval of the version to be published and takes responsibility for the affirmations regarding article submission (eg, not under consideration by another journal), the integrity of the data presented, and the statements regarding compliance with institutional review board/Declaration of Helsinki requirements.

## Supporting information


**Disclosure Form**:

## References

[1] Yang L , Ye T , Liu H , et al. A case of anti‐MDA5‐positive dermatomyositis after inactivated COVID‐19 vaccine. J Eur Acad Dermatol Venereol 2023;37(2):e127–e129. doi:10.1111/jdv.18653 36222733 PMC9874769

[2] Takahashi S , Kato A , Hashimoto K , et al. A case of anti‐melanoma differentiation‐associated gene 5 antibody‐positive dermatomyositis‐associated rapidly progressive interstitial lung diseases developed after administration of COVID‐19 vaccine and subsequent pneumococcal vaccine. Respirol Case Rep 2022;10(12):e01064. doi:10.1002/rcr2.1064 36348741 PMC9630759

[3] Wang S , Noumi B , Malik F , et al. A rare case of MDA‐5‐positive amyopathic dermatomyositis with rapidly progressive interstitial lung disease following COVID‐19 mRNA vaccination ‐ a case report. SN Compr Clin Med 2023;5(1):18. doi:10.1007/s42399-022-01357-0 36530960 PMC9735185

[4] Kitajima T , Funauchi A , Nakajima T , et al. Antimelanoma differentiation‐associated gene 5 antibody‐positive interstitial lung disease after vaccination with COVID‐19 mRNA vaccines. J Rheumatol 2022;49(10):1158–1162. doi:10.3899/jrheum.220259 35705246

[5] Sugimoto T , Yorishima A , Oka N , et al. Appearance of anti‐MDA5 antibody‐positive dermatomyositis after COVID‐19 vaccination. Mod Rheumatol Case Rep 2023;7(1):108–112. doi:10.1093/mrcr/rxac064 35950798

[6] Gonzalez D , Gupta L , Murthy V , et al. Anti‐MDA5 dermatomyositis after COVID‐19 vaccination: a case‐based review. Rheumatol Int 2022;42(9):1629–1641. doi:10.1007/s00296-022-05149-6 35661906 PMC9166182

[7] Holzer MT , Krusche M , Ruffer N , et al. New‐onset dermatomyositis following SARS‐CoV‐2 infection and vaccination: a case‐based review. Rheumatol Int 2022;42(12):2267–2276. doi:10.1007/s00296-022-05176-3 35939078 PMC9358381

[8] Ding Y , Ge Y . Inflammatory myopathy following coronavirus disease 2019 vaccination: a systematic review. Front Public Health 2022;10:1007637. doi:10.3389/fpubh.2022.1007637 36339243 PMC9634642

[9] García‐Bravo L , Calle‐Rubio M , Fernández‐Arquero M , et al. Association of anti‐SARS‐COV‐2 vaccine with increased incidence of myositis‐related anti‐RNA‐synthetases auto‐antibodies. J Transl Autoimmun 2022;5:100160. doi:10.1016/j.jtauto.2022.100160 35789569 PMC9242685

[10] Teijaro JR , Farber DL . COVID‐19 vaccines: modes of immune activation and future challenges. Nat Rev Immunol 2021;21(4):195–197. doi:10.1038/s41577-021-00526-x 33674759 PMC7934118

[11] VA Health Systems Research . Corporate Data Warehouse (CDW). https://www.hsrd.research.va.gov/for_researchers/cdw.cfm

[12] Department of Veterans Affairs Office of Information and Technology . Compensation and Pension Record Interchange (CAPRI) Software Version 2.7 System Administration and Technical Guide. Department of Veterans Affairs; 2023.

[13] Lundberg IE , Tjärnlund A , Bottai M , et al; International Myositis Classification Criteria Project Consortium, the Euromyositis Register, and the Juvenile Dermatomyositis Cohort Biomarker Study and Repository (UK and Ireland). 2017 European League Against Rheumatism/American College of Rheumatology classification criteria for adult and juvenile idiopathic inflammatory myopathies and their major subgroups. Arthritis Rheumatol 2017;69(12):2271–2282. doi:10.1002/art.40320 29106061 PMC5846474

[14] Centers for Disease Control and Prevention . Surveillance and data analytics. US Department of Health and Human Services, Centers for Disease Control and Prevention. January 12, 2025. https://covid.cdc.gov/covid-data-tracker

[15] Diaz‐Menindez M , Sullivan MM , Wang B , et al. Dermatomyositis in association with SARS‐CoV‐2 infection or COVID‐19 vaccine. Arthritis Care Res (Hoboken) 2024;76(1):98–104. doi:10.1002/acr.25236 37728071

[16] David P , Sinha S , Iqbal K , et al. MDA5‐autoimmunity and interstitial pneumonitis contemporaneous with the COVID‐19 pandemic (MIP‐C). EBioMedicine 2024;104:105136. doi:10.1016/j.ebiom.2024.105136 38723554 PMC11090026

[17] Fiorentino DF , Chung LS , Christopher‐Stine L , et al. Most patients with cancer‐associated dermatomyositis have antibodies to nuclear matrix protein NXP‐2 or transcription intermediary factor 1γ. Arthritis Rheum 2013;65(11):2954–2962. doi:10.1002/art.38093 24037894 PMC4073292

[18] Syrmou V , Liaskos C , Ntavari N , et al. COVID‐19 vaccine‐associated myositis: a comprehensive review of the literature driven by a case report. Immunol Res 2023;71(4):537–546. doi:10.1007/s12026-023-09368-2 36928720 PMC10018601

[19] Baden LR , El Sahly HM , Essink B , et al; COVE Study Group . Efficacy and safety of the mRNA‐1273 SARS‐CoV‐2 vaccine. N Engl J Med 2021;384(5):403–416. doi:10.1056/NEJMoa2035389 33378609 PMC7787219

[20] Khoo T , Lilleker JB , Thong BY , et al. Epidemiology of the idiopathic inflammatory myopathies. Nat Rev Rheumatol 2023;19(11):695–712. doi:10.1038/s41584-023-01033-0 37803078

[21] Veterans Health Administration . About VHA. https://www.va.gov/health/aboutvha.asp

[22] Kouranloo K , Dey M , Elwell H , et al. A systematic review of the incidence, management and prognosis of new‐onset autoimmune connective tissue diseases after COVID‐19. Rheumatol Int 2023;43(7):1221–1243. doi:10.1007/s00296-023-05283-9 36786873 PMC9927056

[23] Nune A , Durkowski V , Pillay SS , et al. New‐onset rheumatic immune‐mediated inflammatory diseases following SARS‐CoV‐2 vaccinations until May 2023: a systematic review. Vaccines (Basel) 2023;11(10):1571. doi:10.3390/vaccines11101571 37896974 PMC10610967

